# Comprehensive Experimental Analysis of the Effect of Drilled Material on Torque Using Machine Learning Decision Trees

**DOI:** 10.3390/ma18133145

**Published:** 2025-07-02

**Authors:** Jan Hnátik, Jaroslava Fulemová, Josef Sklenička, Miroslav Gombár, Alena Vagaská, Jindřich Sýkora, Adam Lukáš

**Affiliations:** Department of Machining Technology, Faculty of Mechanical Engineering, University of West Bohemia, Univerzitní 22, 301 00 Plzeň, Czech Republic; jhnatik@fst.zcu.cz (J.H.); fulemova@fst.zcu.cz (J.F.); sklenick@fst.zcu.cz (J.S.); gombar@fst.zcu.cz (M.G.); sykora@fst.zcu.cz (J.S.); alukas@fst.zcu.cz (A.L.)

**Keywords:** torque, drilling, machine learning, decision trees, C45 steel (AISI 1045), case-hardened steel 16MnCr5

## Abstract

This article deals with drilling, the most common and simultaneously most important traditional machining operation, and which is significantly influenced by the properties of the machined material itself. To fully understand this process, both from a theoretical and practical perspective, it is essential to examine the influence of technological and tool-related factors on its various parameters. Based on the evaluation of experimentally obtained data using advanced statistical methods and machine learning decision trees, we present a detailed analysis of the effects of technological factors (*f_n_*, *v_c_*) and tool-related factors (*D*, *ε_r_*, *α*_0_, *ω_r_*) on variations in torque (*M_c_*) during drilling of two types of engineering steels: carbon steel (C45) and case-hardening steel (16MnCr5). The experimental verification was conducted using CTS20D cemented carbide tools coated with a Triple Cr SHM layer. The analysis revealed a significant influence of the material on torque variation, accounting for a share of 1.430%. The experimental verification confirmed the theoretical assumption that the nominal tool diameter (*D*) has a key effect (53.552%) on torque variation. The revolution feed (*f_n_*) contributes 36.263%, while the tool’s point angle (*ε_r_*) and helix angle (*ω_r_*) influence torque by 1.189% and 0.310%, respectively. No significant effect of cutting speed (*v_c_*) on torque variation was observed. However, subsequent machine learning analysis revealed the complexity of interdependencies between the input factors and the resulting torque.

## 1. Introduction

Drilling is a manufacturing operation used to create circular holes in a workpiece, most commonly using a rotating cutting tool, the drill bit [1,2]. Kurt et al. [3] introduced various drilling processes commonly used in the aerospace and automotive industries, including electron beam machining, ultrasonic machining, electrochemical machining, and abrasive jet machining. Despite advancements in non-traditional methods, conventional drilling remains one of the most widely used machining processes [4,5,6]. Rui et al. [7] further emphasized its significant economic importance, as drilling is typically one of the final steps in the production of mechanical components, a point also supported by other studies [8,9]. In the drilling process, tool geometry and plastic deformation introduce significant complexity [1]. During drilling, the cutting speed near the center of the drill approaches zero, while both the cutting speed and rake angle vary with distance from the tool’s center (axis). The cutting edge reaches the highest speed, known as the peripheral cutting speed at the major diameter [1]. Due to these variations, material removal occurs primarily through extrusion. Roteberg et al. [10] found that the drilling process begins at the point of contact between the tool tip and the workpiece, progressing through a penetration phase until full hole formation is achieved. Guibert et al. [11] highlighted that drilling deep, small-diameter holes presents challenges in chip evacuation, often resulting in frequent tool failures and poor hole quality. Typically, cutting tools feature two main edges: the cutting edge and the chisel edge. The removed material undergoes significant plastic deformation due to the indentation and rotation of the chisel edge. Based on experimental findings, Rivero et al. [12] reported that plastic deformation within the shear zone and friction between the tool and the removed material are key sources of heat generation during drilling.

Tsao and Hocheng [13] demonstrated that revolution feed and tool diameter are the most significant factors influencing feed force, while revolution feed and spindle speed have the greatest impact on surface roughness. Heinemann et al. [14] stated that despite extensive research, drilling remains one of the most challenging machining operations, which is also confirmed by many researchers nowadays [15,16,17,18]. A major issue in the drilling process is chip entrapment within the tool’s flutes, which increases friction between the tool and the workpiece and can ultimately lead to a sudden and significant rise in torque [19]. In this context, chip size and shape are influenced by a combination of workpiece material and cutting tool geometry and cutting conditions [20,21,22,23]. Bakkal et al. [24] identified eight fundamental chip formation types based on experimental research. Additionally, the method of chip evacuation from the cutting zone plays a crucial role in determining the final surface quality of the machined hole [25].

Surface roughness plays a crucial role in drilling, influencing how machined surfaces interact with the rest of the assembly [26,27]. It is often a strong predictor of a mechanical component’s performance, as surface irregularities can serve as nucleation sites for cracks or corrosion [28]. Kilickap [29] observed that increasing cutting speed and revolution feed generally leads to greater surface roughness, whereas larger point angles under the same cutting conditions resulted in reduced roughness. Rahman et al. [30] reported that improved drilling performance is associated with lower surface roughness and reduced burr height. They also highlighted that drill diameter, revolution feed, and spindle speed are key factors influencing the final roughness of a drilled hole. As the cutting tool diameter decreases, surface roughness tends to improve, as larger drill diameters remove more material per rotation compared to smaller tools. Kilickap et al. [31] developed an empirical model to analyse the influence of drilling parameters on surface roughness for machining AISI 1045 steel. Modeling and machine learning techniques were also used in [32].

The force conditions in the drilling process are primarily determined by the thrust force, which acts in the axial direction. This thrust force, also referred to as the feed force, operates in the direction of the tool’s feed motion and tends to push the cutting tool away from the workpiece. López de Lacalle et al. [33] stated that feed force and torque result from a combination of two effects: indentation by the chisel edge and material shearing by the main cutting edges. They employed a geometry with a sectioned cutting edge: The secondary cutting edge, formed at the intersection of two lateral surfaces, serves to separate the main cutting edges, enhancing the overall rigidity of the cutting tool and preventing fracture. The main cutting edges are defined by the intersection of the tool’s rake face and lateral surfaces. Arul et al. [34] proposed that feed force can serve as a process indicator to assess the condition of the cutting wedge. Any variation in feed force may indicate changes in the cutting wedge’s state due to deformation, chipping, or other forms of tool wear. Based on experimental results, Basavarajappa et al. [35] found that an increase in revolution feed leads to a rise in feed force across all spindle speed combinations. Sharman et al. [36] defined that as drilling depth increases, torque also rises, accompanied by higher cutting temperatures and increased tool wear. To prevent excessive tool wear and reduce the risk of tool fracture, controlling the cutting (torque) force becomes essential. Jayabal et al. [37] analysed feed force and torque using a full factorial design and reached the same conclusion—revolution feed is the most significant factor influencing feed force and torque. In contrast, drill diameter and cutting speed were found to have an insignificant effect on changes in the feed force component and torque. Singh et al. [38] utilized an artificial neural network to analyse the influence of the feed force component and torque on tool wear progression. Their findings indicate that a larger drill diameter and higher revolution feed lead to increased torque values, whereas a combination of higher spindle speeds and larger drill diameters reduces torque. Conversely, a larger cutting tool diameter combined with lower spindle speeds results in higher feed force values. Young et al. [39] identified drilling depth and revolution feed as the key factors determining torque magnitude. When the drilling depth exceeded five times the tool diameter, a significant increase in torque was observed. This rise in torque elevates tool temperatures in the cutting zone, accelerating tool wear and increasing the risk of tool breakage. Torque is a critical factor influencing machining time, either increasing or decreasing it depending on the cutting conditions. Tsao [40] developed a mathematical model for predicting feed force based on revolution feed, spindle speed, and tool diameter. Additionally, the cutting edge plays a crucial role in increasing feed force. According to Lin [41], curvature of the cutting edge reduces both the feed force component and torque values.

Most researchers [42,43,44] have focused on predicting the feed force component and torque based on variations in cutting speed and tool diameter, primarily because changes in torque can be used to monitor and control tool damage and wear. This study aimed to determine experimentally, using the Design of Experiments methodology [45], the contribution of key technological and selected tool factors to torque variation during the drilling of two structural steels, C45 and 16MnCr5. Additionally, it aimed to examine the influence of the workpiece material on torque variation. To gain deeper insight into the effects of these factors and their interactions, experimentally acquired data were analysed using decision regression trees, a machine learning technique [46,47].

Machine learning (ML) and artificial intelligence tools (AIT) are relatively little used in classical drilling processes (mechanical engineering practice). The situation is different in the research of drilling processes in petroleum engineering [48], or in rock drilling, where there are a relatively large number of review and research studies [49]. Despite the lack of relevant literature, we perceive an increased interest in machine learning algorithms in drilling aluminum alloys [50] for predicting the downforce, aluminum composites [51]. While many studies have analyzed process parameters (forces, torque) in steel drilling using classical methods, the authors present an original approach using ML techniques to analyze the same problem, thereby simultaneously addressing a gap in current research in the field. Extensive experimental work, statistical validation, and implementation of decision trees allowed for a comprehensive and at the same time more detailed analysis of the complex interactions of drilling parameters and their impact on the response (torque). This is the novelty and contribution of the article. On the other hand, we would like to emphasize that the main goal of the article was not the application of the principles of machine learning and artificial intelligence (beyond the framework of traditional statistical methods), but rather a deeper analysis of the drilling process itself.

## 2. Materials and Methodology

### 2.1. Materials Description

The analysis of the influence of material, technological, and tool-related factors on torque variation during drilling was conducted on two types of engineering steels. The first was carbon steel C45 (AISI 1045, EN 1.0503), classified as an engineering steel intended for quenching and tempering. C45 steel is a medium-strength steel with good machinability and excellent tensile properties. It is commonly used as a non-alloyed steel for manufacturing low-stress machine components in either a quenched and tempered or annealed condition. Optimal mechanical properties, including toughness, are achieved in a hardened and subsequently tempered state. For complex-shaped components, oil quenching is preferred to prevent crack formation. From the perspective of machinability, C45 steel is considered a benchmark material, classified with a machinability rating of 14b (or alternatively 13b).

The second examined steel was 16MnCr5 (C10E, EN 10 132-2). This steel is classified as a low alloy manganese–chromium steel specifically designed for case hardening. The 16MnCr5 steel exhibits good hot formability and can also be cold-formed after annealing. It has excellent machinability and weldability. This steel is suitable for mechanical components requiring quenching and tempering up to a diameter of 35 mm and for case-hardening applications where high core strength is essential. Typical applications include shafts, gears, camshafts, piston pins, and gear couplings. The 16MnCr5 was selected as a representative steel for case-hardening applications. The chemical composition of the material (C45; 16MnCr5) used in experiment is presented in Table 1.

**Figure 1:**
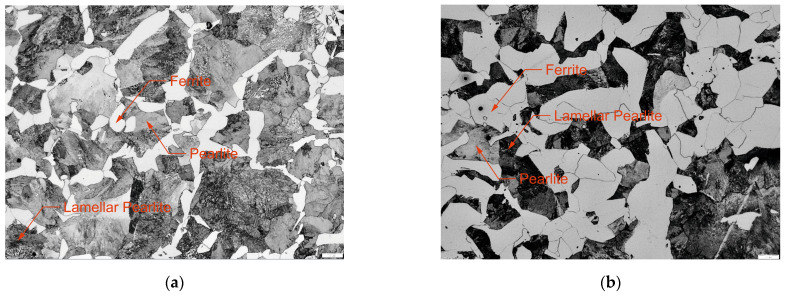
The microstructure of the machined material (**a**) C45; (**b**) 16MnCr5.

Another evaluated property of the experimental workpiece material was the ultimate tensile strength (R_m_), determined through a tensile test conducted on six specimens of standardized shape and dimensions. For the first material C45, an average R_m_ value of 740.500 ± 1.445 MPa was recorded with a minimum (maximum) value of 738 MPa (742 MPa). An average R_m_ value of 549.168 ± 10.895 MPa was determined for 16MnCr5. We were also interested in the hardness value (HV10); the value of 221.500 ± 3.023 was recorded for steel C45 and 175.667 ± 2.268 for 16MnCr5 steel. The microstructure of C45 steel (Figure 1a) exhibits a ferritic–pearlitic structure, with a ferrite content of 17.488 ± 1.291%. Similarly, the microstructure of 16MnCr5 steel (Figure 1b) also features a ferritic–pearlitic structure, but with a significantly higher ferrite content of 53.213 ± 1.975%.

### 2.2. Equipment Used and Experimental Procedure

**Figure 2:**
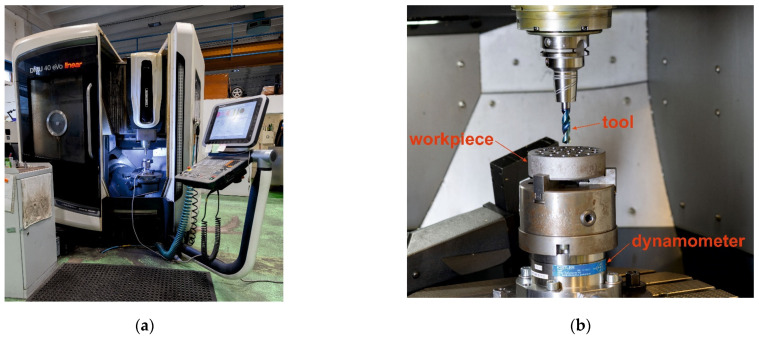
Experimental setup: (**a**) DMU eVo 40 linear machine; (**b**) detailed view of the workpiece clamping, tool, and dynamometer in the machine.

The experiment was conducted using a DMU eVo 40 linear machine (DMG, Bielefeld, Germany), shown in Figure 2a, produced by DMG MORI CO., Ltd., equipped with a hydraulic tool holder with interchangeable sleeves for tool diameters ranging from 8.0 mm to 12.0 mm. The DMU eVo series consists of simultaneous 5-axis machines featuring NC-controlled automatic rotary tables. These models utilize a bridge-type construction, ensuring high rigidity and long-term machining stability. The DMU 40 eVo is equipped with a 450 mm × 400 mm worktable capable of supporting loads of up to 250 kg. This model is suitable for machining small- to medium-sized workpieces across various industries, including medical components, automotive, and aerospace applications. The measurement of the investigated response variable (torque) during the machining process was performed using a KISTLER 9272 dynamometer (Kistler, Winterthur, Switzerland). The workpiece was secured to the dynamometer’s contact surface using a three-jaw chuck seen in Figure 2b. The workpiece was a ground cylindrical bar with dimensions Ø118 mm × 30 mm, made of C45 and 16MnCr5 materials, respectively.

### 2.3. Cutting Tool and Technological Factors

The drill bit parameters considered as controlled variables (factors) in the experiment are restricted to its macrogeometry as seen in Table 2. All other tool parameters, including microgeometry and surface characteristics, are treated as constant factors. To ensure the numerical and statistical validity of the experimental results, it is essential that the controlled factors remain mutually independent. Adhering to this fundamental condition served as the basis for classifying the tool-related factors into controlled and constant categories.

**Figure 3:**
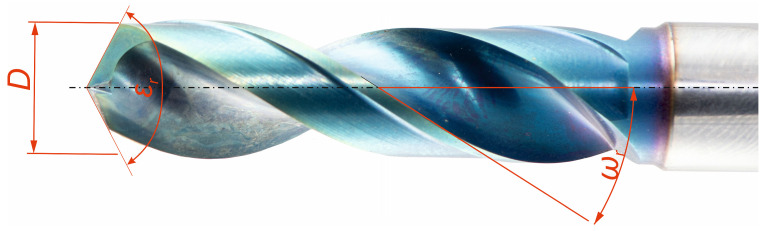
Controlled cutting tool factors.

A schematic representation of the controlled tool factors (*D*—nominal tool diameter [mm], *ω_r_*—helix angle [°], *ε_r_*—point angle [°]) is presented in Figure 3.

The factors that were maintained at a constant level throughout the experiment and across all individual trials are as follows: tool flank angle in the orthogonal plane (*α*_0_ = 10°); tool core diameter 0.3*D* [mm]; flute facet width 0.115*D* [mm]; flank facet width 0.135*D* [mm]; cutting edge radius 15 [µm]; drilling depth 2*D* [mm]; chisel edge length 0.025*D* [mm]. The primary technological factors that were systematically varied in a controlled manner during the experiment were the following: feed per revolution *f_n_* [mm·rev^−1^]; cutting speed *v_c_* [m/min]. The levels of the varied tool and technological factors are presented in Table 2. Each experimental trial was repeated 10 times to ensure reliable results while accounting for measurement error analysis. In total, 1500 individual experiments were conducted.

**Figure 4:**
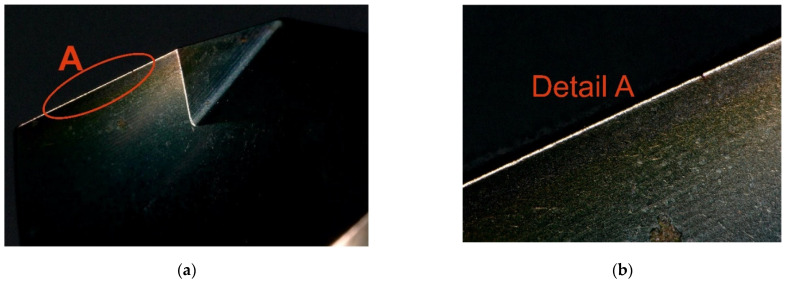
Condition of the new cutting tool: (**a**) Condition of the rake face; (**b**) detailed view of the rake face.

**Figure 5:**
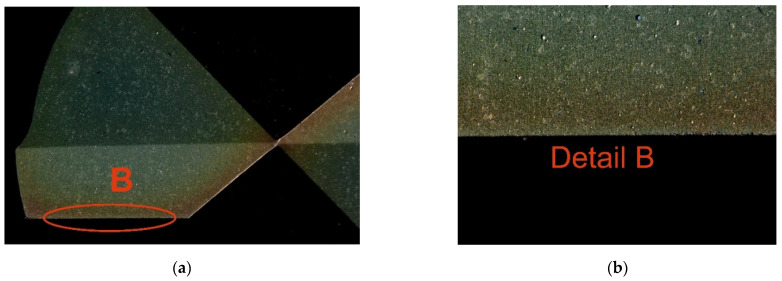
Condition of the new cutting tool: (**a**) Condition of the flank face; (**b**) detailed view of the flank face.

**Figure 6:**
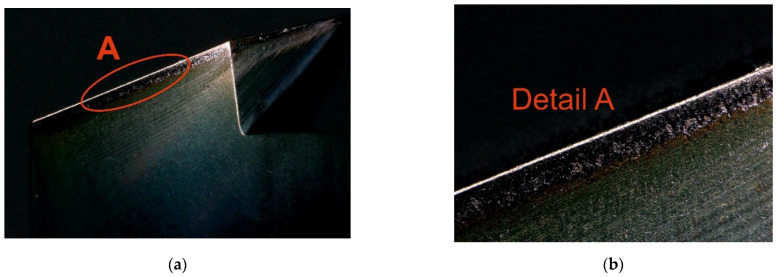
Cutting tool wear after drilling under the following conditions: *D* = 10.0 mm, *ε_r_* =137.5°, *ω_r_* = 30.0 °, *f_n_* = 0.175 mm·rev^−1^, *v_c_* = 115.0 m·min^−1^: (**a**) Wear on the rake face of the tool; (**b**) detailed view of rake face wear.

**Figure 7:**
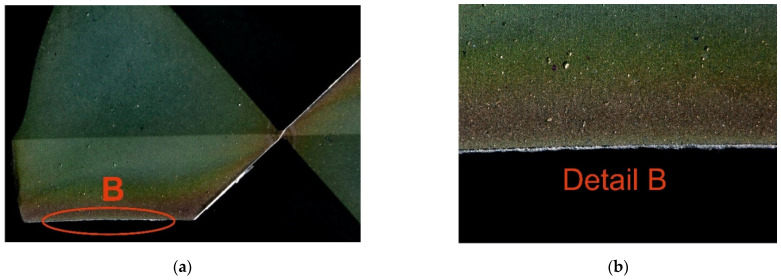
Cutting tool wear after drilling under the following conditions: *D* = 10.0 mm, *ε_r_* =137.5°, *ω_r_* = 30.0 °, *f_n_* = 0.175 mm·rev^−1^, *v_c_* = 115.0 m·min^−1^: (**a**) Wear on the flank face of the tool; (**b**) detailed view of flank face wear.

The cutting fluid used in the experiment was BLASOCUT BC 35 KOMBI, an oil emulsion from Blaser Swisslube CZ. The concentration was set to 6.9%, within the manufacturer’s recommended range of 6% to 8%. The drill bits were made of CTS20D (Ceratizit, Mamer, Luxembourg) and coated with a Triple Cr SHM layer, featuring a slightly convex cutting edge geometry. To avoid distorting the measured response (torque) due to tool wear, a new cutting tool was used for each trial. However, it is worth noting that tool wear after the experiment was minimal compared to a new tool, as shown in Figure 4 and Figure 5, and further documented in Figure 6 and Figure 7.

### 2.4. Machine Learning and Decision Trees

The influence of selected technological and tool factors on the torque requires a comprehensive approach; therefore, in order to achieve the set research goal, we applied machine learning (ML) methods in the next step of the methodological procedure; specifically, decision trees, while the CERT method (Computer Emergency Response Team) was chosen for tree construction [33,52,53]. Machine learning (ML) has played a key role in solving complex problems and has significantly advanced various areas of our lives. Methods based on decision trees (DT) have gained considerable popularity among a wide range of ML algorithms due to their simplicity and interpretability [52]. With advances in technology and the availability of large data sets, machine learning algorithms are becoming increasingly powerful and accurate in making predictions and informed decisions. These applications are transforming the way various processes (including organizations) are managed and are the basis for a more efficient and data-driven future [53]. Decision tree-based algorithms have various advantages (ability to process categorical and numerical data, interpretability, transparency) and many applications [54]. The basic idea of DT algorithms is that they recursively divide a data set into homogenous subsets based on specified rules (the defined attributes) until a stopping criterion is met. The result of this process is a tree structure where each node represents a decision or division based on a specific attribute [55]. Although there are many DT algorithms, they all have a similar structure. The creation of a decision tree consists of three parts: division rules, stopping criteria, and assignment rules [55]. The root node, which is the starting point of the tree, represents the entire data set. The algorithm identifies the element and threshold value that leads to the best division based on a specific criterion. The process continues recursively, with each subset of the data being further divided at each child node [54]. This continues until a stopping criterion is reached. The nodes where the tree ends (the end or terminal node) represent the results or class labels. The division decision at each node is made using mathematical formulations such as information gain, Gini impurity, or variance reduction [56]. In accordance with the facts mentioned above and based on experimental measurements, a suitable decision tree was constructed.

## 3. Results and Discussion

**Figure 8:**
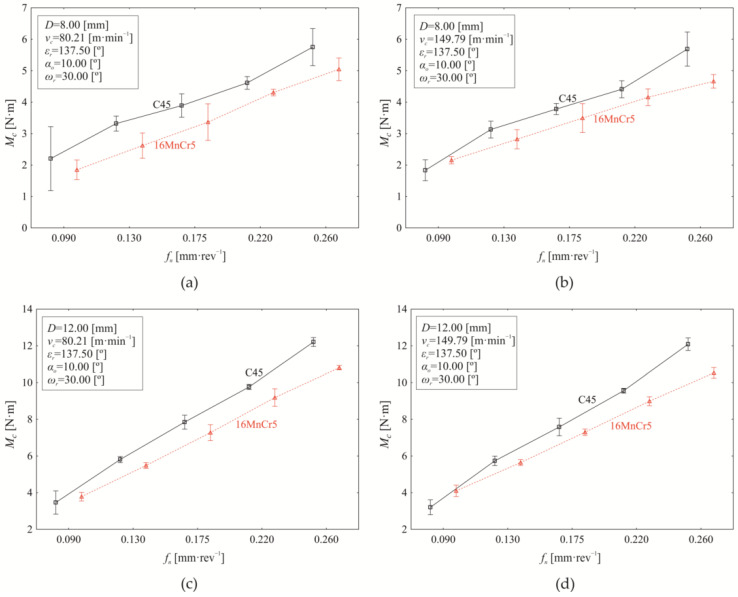
Dependence of torque *M_c_* on revolution feed *f_n_* at constant values of *ε_r_* = 137.5°, α_o_ = 10.00°, *ω_r_* = 30.0° while simultaneously varying nominal diameter *D* and cutting speed *v_c_* as follows: (**a**) *D* = 8.00 mm, *v_c_* = 80.21 m·min^−1^; (**b**) *D* = 8.00 mm, *v_c_* = 149.79 m·min^−1^; (**c**) *D* = 12.00 mm, *v_c_* = 80.21 m·min^−1^; (**d**) *D* = 12.00 mm, *v_c_* = 149.79 m·min^−1^.

Machining processes generally are multi-factorial stochastic processes, where the individual factors of the machine-tool-workpiece-fixture system are further influenced by the technological conditions of machining. Moreover, the input factors do not act solely as main effects but also exhibit significant mutual interactions. The variation in the examined response (torque *M_c_*) as a function of revolution feed (*f_n_*) when setting constant values of *ε_r_* = 137.50°, *α_o_* = 10.00°, *ω_r_* = 30.00° while simultaneously varying the nominal tool diameter (*D*) and cutting speed (*v_c_*) for both investigated materials (C45, 16MnCr5) is presented in Figure 8.

As shown in Figure 8, increasing the revolution feed generally leads to a corresponding increase in torque (*M_c_*). At a nominal tool diameter of *D* = 8.00 mm and a cutting speed of *v_c_* = 80.21 m·min^−1^, see Figure 8a, the material’s influence on torque variation is 4.520%. Since machining processes are inherently stochastic, and every measurement is subject to errors (gross, systematic, and random), mathematical statistical tools were used to ensure the accuracy of the results and the reliability of the conclusions drawn.

The influence of machined material (4.520%) on torque variation under the experimental conditions shown in Figure 8a is statistically significant (*p* < 0.000) at the chosen significance level of α = 0.05. The dominant factor affecting torque variation (Figure 8a) is revolution feed, accounting for 91.450% of the effect (*p* < 0.000). Although relatively small (0.470%), the interaction between revolution feed and material also has a statistically significant effect on the measured response (*M_c_*) (*p* = 0.023). Additionally, Figure 8a indicates that the mean torque during drilling of C45 steel (3.956 ± 0.352 Nm) is higher than that of 16MnCr5 steel (3.437 ± 0.332 Nm) across the tested revolution feed (*f_n_*) range. The difference in mean torque values between S45 and 16MnCr5 is 0.519 ± 0.096 Nm as evaluated using Scheffé’s test significant (*p* < 0.000). However, the revolution feed affects the difference in mean torque values between drilling C45 and 16MnCr5 (Figure 8a), making it statistically significant only at revolution feeds of 0.130 mm·rev^−1^ (*p* < 0.000), 0.175 mm·rev^−1^ (*p* = 0.001), and 0.260 mm·rev^−1^ (*p* < 0.000).

Increasing the cutting speed to *v_c_* = 149.79 m·min^−1^ while maintaining *D* = 8.00 mm (Figure 8b) reduces the influence of the material on the torque to 1.900% (*p* < 0.000). In contrast, the effect of the revolution feed slightly increases to 92.720% (*p* < 0.000), while the interaction between revolution feed and material shows a significant rise to 3.580% (*p* < 0.000) compared to the conditions listed in Figure 8a. The difference in mean torque values (0.311 ± 0.064 Nm) between drilling C45 steel (3.769 ± 0.372 Nm) and 16MnCr5 steel (3.458 ± 0.261 Nm) is statistically significant (*p* < 0.000) when setting factors at levels presented in Figure 8b; the dominant influence of the revolution feed is again evident. Significant differences in mean torque (between C45 and 16MnCr5) are observed at *f_n_* of 0.090 mm·rev^−1^ (−0.321 ± 0.241 Nm, *p* < 0.000), at 0.130 mm·rev^−1^ (0.309 ± 0.103 Nm, *p* < 0.000), and at the upper limit of the experimental range of *f_n_* = 0.260 mm·rev^−1^ (1.027 ± 0.136 Nm, *p* < 0.000). When using drill of *D* = 8.00 mm, increasing the cutting speed from 80.21 m·min^−1^ (Figure 8a) to 149.79 m·min^−1^ (Figure 6b) reduces the mean torque value by 4.948% (*p* < 0.000) during machining of C45 steel. In contrast, for 16MnCr5 steel, the torque value shows a slight increase of 0.599%, which is not statistically significant (*p* = 0.968).

When setting drilling conditions as listed in Figure 8c, with increasing the nominal drill diameter to *D* = 12.00 mm (at *v_c_* = 80.21 m·min^−1^), the material effect on torque (*M_c_*) reduces to 0.83% (*p* < 0.000), the revolution feed effect on *M_c_* increases to 97.860% (*p* < 0.000), while the effect of their interaction (material and revolution feed) rises to 0.970% (*p* < 0.000). Moreover, increasing the drill diameter *D* from 8 mm to 12.00 mm at a cutting speed of 80.21 m·min^−1^ leads to an increase in mean torque by 97.559% for C45 steel (by 112.598% for 16MnCr5 steel). Significant differences in *M_c_* mean values between C45 and 16MnCr5 were observed at revolution feeds higher than 0.090 mm·rev^−1^; specifically, at 0.130 mm·rev^−1^ (0.327 ± 0.058 Nm, *p* < 0.000), 0.175 mm·rev^−1^ (0.572 ± 0.146 Nm, *p* < 0.000), 0.220 mm·rev^−1^ (0.579 ± 0.111 Nm, *p* < 0.000), and 0.260 mm·rev^−1^ (1.398 ± 0.065 Nm, *p* < 0.000).

On increasing the cutting speed *v_c_* to 149.79 m·min^−1^ when using drill of D = 12.00 mm (Figure 8d), the material effect on torque variation reduces to 0.350% (*p* < 0.000), while the contribution of revolution feed to the measured response (*M*_c_) remains dominant at 97.240% (*p* < 0.000). At the same time, the influence of interaction (material and revolution feed) increases to 2.130% (*p* < 0.000) compared to the conditions in Figure 8c. Comparison analysis of *M_c_* mean values (Figure 8c,d), while setting constant drill diameter (*D* = 12.00 mm) and varying cutting speed (*v_c_* = 80.21 m·min^−1^, *v_c_* = 149.79 m·min^−1^), indicates that increasing the cutting speed *v_c_* decreases the mean torque value by 2.424% (*p* < 0.000) for C45 steel. On the other hand, the mean torque value increases slightly by 0.053% (*p* = 0.999) with increasing of *v_c_* for 16MnCr5 steel, but it is not statistically significant.

**Figure 9:**
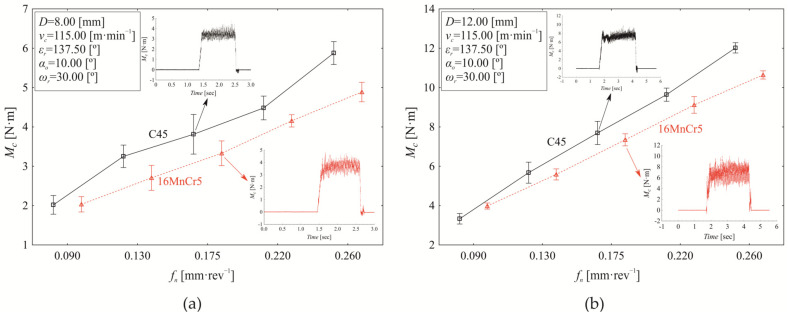
Torque variation (*M*_c_) as a function of revolution feed (*f_n_*) at *ε_r_* = 137.5°, α_o_ = 10.00°, *ω_r_* = 30.0°, and *v_c_* = 115.00 m·min^−1^ for (**a**) *D* = 8.00 mm and (**b**) *D* = 12.00 mm.

In the torque analysis presented in Figure 8, we focused on the boundary values of the selected controlled technological (cutting speed) and tool-related (nominal tool diameter) factors while varying the revolution feed. Since the analysis confirmed a significant influence of revolution feed, material, and their interaction on torque variation at the selected boundary points (*v_c_*, *D*), we aim to refine the analysis further. This will be done by examining the mean cutting speed values at a nominal drill diameter *D* = 8.00 mm (Figure 9a) and at the upper limit of the tested interval, *D* = 12.00 mm (Figure 9b).

The variation in torque as a function of revolution feed at a cutting speed of *v_c_* = 115.00 m·min^−1^ and a nominal tool diameter of *D* = 8.00 mm is shown in Figure 9a. Under these defined technological and tool-related conditions, the influence of material on torque variation is 3.860% (*p* < 0.000), the influence of revolution feed is 92.830% (*p* < 0.000), and the influence of the interaction between material and revolution feed is 1.890% (*p* < 0.000). Differences in mean torque values between drilling C45 and 16MnCr5 vary depending on the applied revolution feed. Statistically significant differences in mean torque values were observed at revolution feeds as follows: *f_n_* = 0.130 mm·rev−^1^ (0.547 ± 0.108 Nm, *p* < 0.000); *f_n_* = 0.175 mm·rev^−1^ (0.483 ± 0.146 Nm, *p* < 0.000); *f_n_* = 0.220 mm·rev^−1^ (0.326 ± 0.082 Nm, *p* < 0.000); and *f_n_* = 0.260 mm·rev^−1^ (0.991 ± 0.096 Nm, *p* < 0.000). Increasing the nominal drill diameter from 8.00 mm (Figure 9a) to *D* = 12.00 mm at cutting speed *v_c_* = 115.00 m·min^−1^ (Figure 9b) results in a 97.276% increase in torque *M*_c_ for C45 steel and 113.074% for 16MnCr5 steel. At this tool diameter (12.00 mm, Figure 9b), the influence of material on torque variation is 0.380% (*p* < 0.000), while the revolution feed contributes 97.780% (*p* < 0.000), and the interaction between material and revolution feed accounts for 1.440% (*p* < 0.000). When considering the combined effect of changing the nominal tool diameter (comparing Figure 9a,b), the overall influence of material on the torque variation is 0.500% (*p* < 0.000), while the revolution feed contributes 45.360% (*p* < 0.000), and the nominal tool diameter accounts for 45.480% (*p* < 0.000). These results highlight that the contribution of technological and tool-related factors to torque variation is strongly dependent on their specific values.

**Figure 10:**
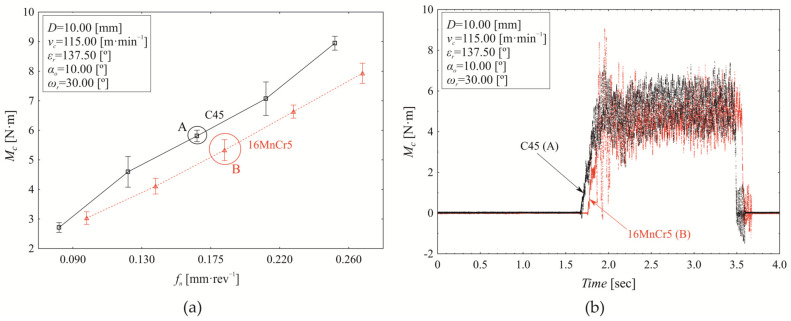
Torque variation (*M*_c_) as a function of revolution feed (*f_n_*) at *ε_r_* = 137.5°, α_o_ = 10.00°, *ω_r_* = 30.0°, *v_c_* = 115.00 m·min^−1^ and (**a**) *D* = 10.00 mm; (**b**) *D* = 10.00 mm, time-domain signal of torque (*M*_c_) recorded during the drilling process at the central revolution feed.

The variation in torque as a function of revolution feed at the mean cutting speed (*v*_c_ = 115.00 m·min^−1^) and mean nominal tool diameter (*D* = 10.00 mm) is shown in Figure 10a,b. The contribution of material to torque variation is 1.150% (*p* < 0.000), while revolution feed accounts for 97.010% (*p* < 0.000), and the interaction between material and revolution feed contributes 1.190% (*p* < 0.000). The mean difference in torque values between drilling C45 steel (5.826 ± 0.611 Nm) and 16MnCr5 steel (5.406 ± 0.502 Nm) is 0.420 ± 0.278 Nm (*p* < 0.000). Additionally, significant differences in torque values were observed between C45 and 16MnCr5 at all applied revolution feed values.

**Figure 11:**
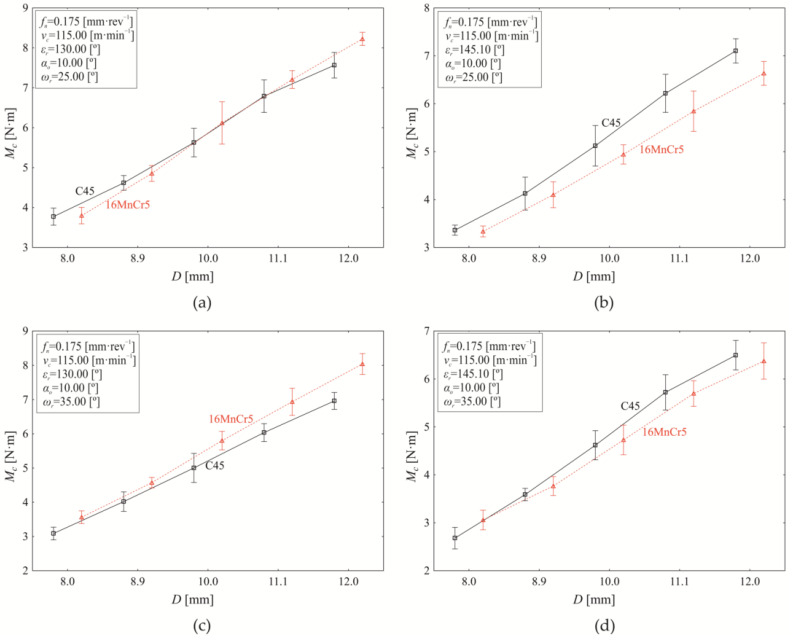
Torque variation (*M_c_*) as a function of tool diameter (*D*) at *f_n_* = 0.175 mm·rev^−1^, *v_c_* = 115.00 m·min^−1^, and *α*_0_ = 10.00°, for (**a**) *εᵣ* = 130.00°, *ωᵣ* = 25.0°; (**b**) *εᵣ* = 145.10°, *ωᵣ* = 25.0°; (**c**) *εᵣ* = 130.00°, *ωᵣ* = 35.0°; (**d**) *εᵣ* = 145.10°, *ωᵣ* = 35.0°.

In the following section, the influence of selected tool factors (*D*, *ε*ᵣ, *ω*ᵣ) on torque variation (*M_c_*) is analysed. As seen in Figure 11, a constant cutting speed of 115.00 m·min^−1^ and a revolution feed of 0.175 mm·rev^−1^ were maintained across all experimental trials, the nominal drill diameter (*D*) was used as the independent, controlled tool factor.

Based on the analysis presented in Figure 11, the experimental results demonstrate that increasing the nominal tool diameter (*D*) leads to a corresponding increase in torque (*M_c_*). A statistically significant material effect on the torque is observed at the point angle of *εᵣ* = 130.00° and helix angle of *ωᵣ* = 25.0° (Figure 11a); its value represents 1.480% at the chosen significance level of α = 0.05. The dominant factor affecting torque variation is the nominal tool diameter (*D*), contributing 97.090% (*p* < 0.000). Additionally, the interaction of material and tool diameter has a statistically significant effect (*p* < 0.000), accounting for 0.520% of the variation. When analysing only the mean torque values for the tested materials, independent of tool diameter, the difference in mean torque (*M_c_*) between C45 steel (5.674 ± 0.399 Nm) and 16MnCr5 steel (6.041 ± 0.457 Nm) is statistically significant (*p* < 0.000) based on Scheffé’s test. However, when considering the effect of nominal tool diameter (*D*), significant differences in torque values appear only at tool diameters of 10.00 mm and above. At *D* = 10.00 mm, the statistical difference in mean torque between C45 (5.627 ± 0.127 Nm) and 16MnCr5 (6.120 ± 0.189 Nm) is −0.493 ± 0.158 Nm, and is statistically significant, *p* < 0.000). This trend is also observed for *D* = 11.10 mm (−0.417 ± 0.113 Nm, *p* < 0.000), and *D* = 12.00 mm (−0.660 ± 0.087 Nm, *p* < 0.000). With the increase of the point angle to *εᵣ* = 145.10° while maintaining a helix angle of *ωᵣ* = 25.0° (Figure 11b), the influence of material on torque variation decreases to 0.700%, compared to Figure 11a. At the same time, the influence of nominal tool diameter increases to 97.580%, while the interaction effect between material and tool diameter decreases to 0.490%. With the increase of the point angle to *εᵣ* = 145.10° while maintaining the helix angle at *ωᵣ* = 25.0° (Figure 11b compared to Figure 11a), the material effect on *M_c_* decreases to 0.700%, nominal tool diameter increases to 97.580%, while the interaction effect (material and drill diameter) decreases to 0.490%.

All evaluated input factors demonstrate a statistically significant effect on torque *M_c_* (*p* < 0.000). However, significant differences in mean torque values (*p* < 0.000) between C45 steel (*M*_c_ = 6.217 ± 0.142 Nm) and 16MnCr5 steel (*M*_c_ = 0.373 ± 0.222 Nm) during drilling at a point angle of *ε_r_* = 145.10° appear only at a nominal tool diameter of *D* = 11.10 mm (resulting torque difference is 0.373 ± 0.222 Nm). At a nominal tool diameter of *D* = 12.00 mm, the resulting torque difference is 0.470 ± 0.090 Nm when comparing *M_c_* during drilling steel C45 (*M_c_* = 7.014 ± 0.090 Nm) and steel 16MnCr5 (6.634 ± 0.089 Nm). Compared to the previous case in Figure 11a, the torque values are higher during drilling steel C45. Increasing the helix angle to *ω_r_* = 35.0° at a point angle settled at *ε_r_* = 130.00° (Figure 11c), the material effect on torque variation increases to 6.040% (*p* < 0.000), the drill diameter effect decreases to 92.690% (*p* < 0.000), and the influence of interaction “material and tool diameter” increases slightly to 0.510% (*p* < 0.000). The difference in mean torque values between C45 and 16MnCr5 steel increases with the nominal tool diameter, ranging from −0.476 ± 0.066 Nm at *D* = 8.00 mm to −1.080 ± 0.099 Nm at *D* = 12.00 mm. It is important to note that all differences in mean torque values for all tested nominal tool diameters are statistically significant (*p* < 0.000). Additionally, torque values during C45 drilling remain lower than those for 16MnCr5 drilling across all cases. Changing the point angle to *ε_r_* =145.10° while maintaining a helix angle of *ω_r_* = 35.0° (Figure 11d) results in a decrease in the influence of material on the torque variation to 0.160%. At the same time, the influence of the nominal tool diameter increases to 98.37%, while the influence of the interaction between material and tool diameter decreases to 0.430%. All contributing factors significantly affect torque variation (*p* < 0.000). However, significant differences in mean torque values between C45 steel and 16MnCr5 steel are observed only at a nominal tool diameter of D = 8.00 mm, with a difference of −0.377 ± 0.077 Nm.

Taking into account all partial experiments carried out under conditions shown in Figure 11, while keeping constant revolution feed and cutting speed (*f_n_* = 0.175 mm·rev^−1^, *v_c_* = 115.00 m·min^−1^), the nominal tool diameter *D* is observed to be the most influential factor (87.390% share of influence in the torque variation, *p* < 0.000). The second most significant controlled factor is the point angle, contributing 6.470% (*p* < 0.000), followed by the helix angle with an impact of 2.110% (*p* < 0.000). Although material as a main effect contributes only 0.740% to torque variation, its influence remains statistically significant (*p* < 0.000). Additionally, several interaction effects significantly influence torque variation (*p* < 0.000), including material and point angle (1.090%), material and helix angle (0.360%), nominal tool diameter and point angle (0.480%), and the combined interaction of material, nominal tool diameter, and point angle (0.420%). For the first significant interaction (material and point angle), a statistically significant difference in mean torque values between C45 steel and 16MnCr5 steel is observed only at *ε_r_* = 130.00°, with a difference of −0.564 ± 0.302 Nm (*p* < 0.000). Regarding the second significant interaction (material and helix angle), statistically significant differences in mean torque values between C45 steel and 16MnCr5 steel are present at both tested helix angles. At *ω_r_* = 25.0°, the difference in mean torque values is −0.076 ± 0.021 Nm (*p =* 0.004), while at *ω_r_* = 35.0°, it increases to −0.433 ± 0.024 Nm (*p* < 0.000).

**Figure 12:**
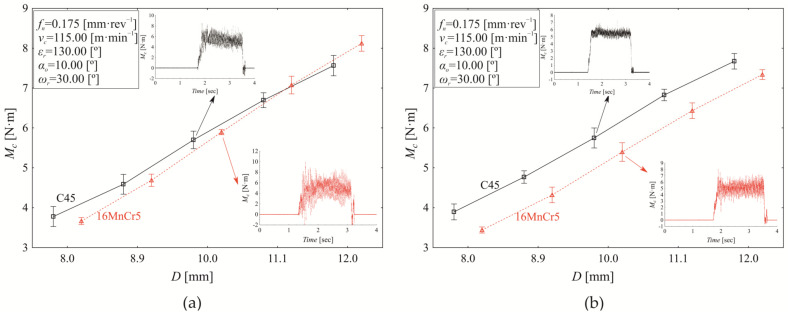
Torque variation (*M_c_*) as a function of nominal tool diameter (*D*) at *f_n_* = 0.175 mm·rev^−1^, *v_c_* = 115.00 m·min^−1^, *α*_0_ = 10.00°, *ω_r_* = 30.0°, and (**a**) *ε_r_* = 130.00°, (**b**) *ε_r_* = 137.50°.

The graphical outputs of analysis of experiments carried out at the borderline factor levels (Table 2) at constant *f_n_* = 0.175 mm·rev^−1^ and *v_c_* = 115.00 m·min^−1^ are shown in Figure 12. The experimental results demonstrate that increasing the nominal tool diameter (*D*) leads to a corresponding increase in torque (*M_c_*).

At a helix angle of *ωᵣ* = 30.0° and a point angle of *εᵣ* = 130.00° (Figure 12a), material accounts for 0.560% of the variation in torque, while the nominal tool diameter contributes 98.450%. The interaction between material and tool diameter has a minor yet statistically significant influence of 0.90% (*p* < 0.000). Statistically significant differences in torque (*p* < 0.000) between C45 and 16MnCr5 begin to emerge at a nominal tool diameter of *D* = 10.00 mm, with a torque difference of −0.196 ± 0.051 Nm. As the nominal tool diameter increases to *D* = 11.10 mm, the difference grows to -0.380 ± 0.073 Nm, reaching −0.557 ± 0.081 Nm at the upper limit of the tested range (*D* = 12.00 mm). Lower values of *M_c_* are achieved when drilling C45 material compared to steel 16MnCr5. With the increase of point angle at *ε_r_* = 137.50° (Figure 12b), the material effect conditionally increases to 2.100% (*p* < 0.000), and the drill diameter effect decreases to 97.580%. As seen in Figure 12b, the influence of “material and drill diameter” interaction is not observed to be significant (*p* = 0.141). When comparing *M_c_* values at experimental conditions defined in Figure 12a,b, there is a significant difference (*p* < 0.000) in the mean torque values (0.193 ± 0.020 Nm). It can be stated that increasing the point angle by 5.769% will result in a reduction of the conditional *M_c_* value by 3.358%, but this conclusion applies only to the conditions shown in Figure 12. The main effect of *ε_r_* on *M_c_* values represents 0.440% (*p* < 0.000). Significant interactions were observed as follows: point angle and material (1.440%), point angle and tool diameter (0.130%), and point angle and material and tool diameter (0.110%).

**Figure 13:**
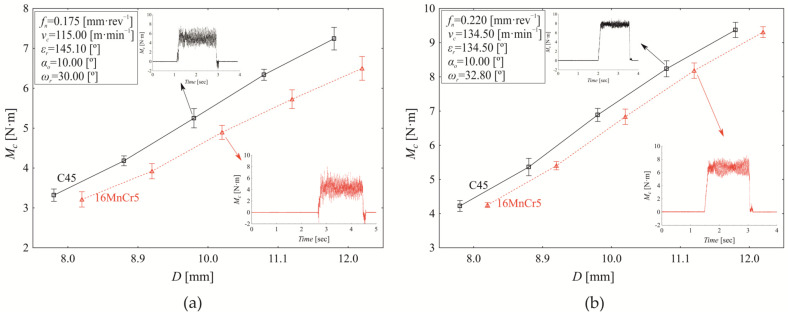
Main effect plot of drill diameter (*D*) on the torque variation (*M_c_*) at *α*_0_ = 10.00° when setting factors as follows: (**a**) *f_n_* = 0.175 mm·rev^−1^, *v_c_* = 115.00 m·min^−1^, *ε_r_* = 145.10°, *ω_r_* = 30.0°; (**b**) *f_n_* = 0.220 mm·rev^−1^, *v_c_* = 134.50 m·min^−1^, *ε_r_* = 134.50 °, *ω_r_* = 23.80°.

The extension analysis of torque dependence on the tool diameter is shown in Figure 13; the point angle is increased to the level of *ε_r_* = 145.10°. At this point angle, we can observe a significant effect of material on torque variation (2.450%, *p* < 0.000); the significant tool diameter effect contributes 96.210% (*p* < 0.000). Additionally, the interaction “material and tool diameter” accounts for 0.770% (*p* < 0.000). When considering the combined conditions from Figure 10a,b and Figure 11a, which represent the effect of the point angle in the range of *ε_r_* = 130.00° to 145.10° while keeping *ω_r_* = 30.0°, *f_n_* = 0.175 mm·rev^−1^, and *v_n_* = 115.00 m·min^−1^ constant, the influence of the point angle on torque variation is 4.440% (*p* < 0.000). In this case, the influence of material decreases to 0.460%, while statistically significant interactions emerge between point angle and material (1.060%) and between point angle and nominal tool diameter (0.280%). When setting constant values of *ω_r_* = 30.0°, *f_n_* = 0.175 mm·rev^−1^, *v_c_* = 115.00 m·min^−1^, while simultaneously increasing point angle *ε_r_* within the interval from *ε_r_* = 130.00° to 145.10° (Figure 12 and Figure 13a), the effect of the point angle (*ε_r_*) on the Mc torque variation represents 4.440% (*p* < 0.000). In this case, the influence of material decreases to 0.460%, while statistically significant interactions emerge between point angle and material (1.060%) and between point angle and nominal tool diameter (0.280%).

Under drilling conditions at level 4, as defined in Table 2, the nominal tool diameter is the only statistically significant factor (*p* < 0.000) influencing torque variation (99.730%). As the nominal tool diameter increases, the mean torque value rises, ranging from 4.237 ± 0.029 Nm at *D* = 8.00 mm to 9.340 ± 0.047 Nm at *D* = 12.00 mm. According to the conditions in Figure 13a, an increase of 0.10 mm in nominal tool diameter results in a 3.011% rise in relative torque value.

The presented analyses highlight an important aspect—the complexity of the drilling process. Its evaluation must consider not only the influence of the material but also the combined effects of tool and technological factors on torque variation. This influence is highly dependent on the specific values of input variables while also being significantly affected by their interactions, which play a crucial role in determining the overall torque behaviour. A comprehensive evaluation of the experiment, which consisted of 1500 individual trials focusing on the influence of selected tool and technological factors on torque variation (M_c_), leads to the conclusion that the overall effect of material on torque variation is 1.430%. Although this influence is relatively low, it remains statistically significant (*p* < 0.000) at a significance level of α = 0.05. This indicates that despite its minor contribution, the material must be considered in the torque analysis. The dominant factor affecting torque variation is the nominal tool diameter, contributing 53.552% (*p* < 0.000), followed by the feed rate, which accounts for 36.263% (*p* < 0.000). Among the other tool-related factors, the point angle influences torque variation by 1.189% (*p* < 0.000), while the helix angle contributes 0.310% (*p* < 0.000). The effect of cutting speed on torque variation was not confirmed (*p* = 0.354) in this analysis.

**Figure 14:**
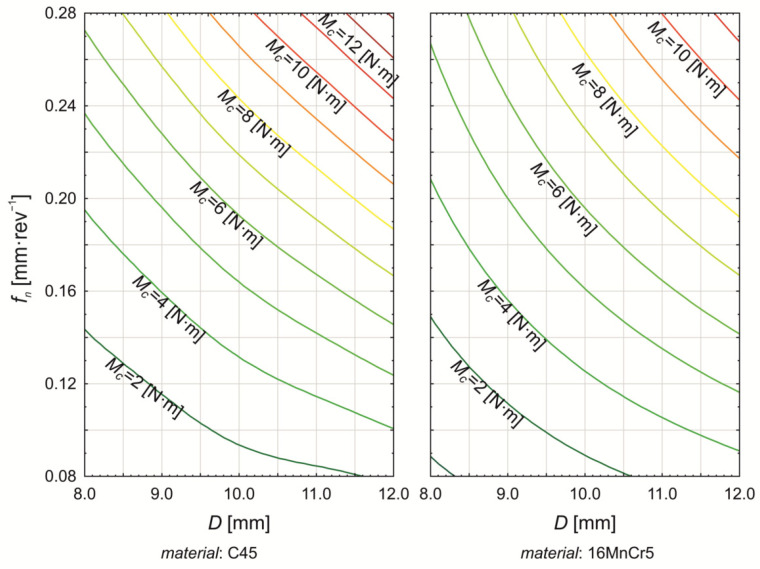
Graphical plots of torque value variation in dependence of simultaneous changes in nominal drill diameter and revolution feed.

The differences between C45 steel and 16MnCr5 steel can be observed in the percentage contribution of tool and technological factors to torque variation. For C45 steel, the nominal tool diameter (*D*) contributes 50.115% (*p* < 0.000) to torque variation, while the feed rate (*f_n_*) accounts for 41.603% (*p* < 0.000). The point angle influences torque variation by 0.486% (*p* < 0.000), and the helix angle contributes 0.680% (p < 0.000). For 16MnCr5 steel, the influence of nominal tool diameter increases to 60.373% (*p* < 0.000), while the contribution of feed rate decreases to 31.480% (*p* < 0.000) compared to C45. Simultaneously, the effect of point angle increases to 2.562% (*p* < 0.000). The helix angle has a minor influence on torque variation for 16MnCr5, accounting for 0.098% (*p* = 0.002). For both materials, the cutting speed does not have a statistically significant effect on torque variation. The influence of the two most significant factors, nominal tool diameter (*D*) and feed rate (*f_n_*), on torque variation in C45 and 16MnCr5 drilling is presented in Figure 14.

Figure 14 illustrates that the torque variation during drilling of C45 and 16MnCr5 steels, as a function of nominal tool diameter and feed rate, follows a similar trend. However, differences are evident in the absolute torque values for each material. Additionally, a gradual shift in the torque dependency on nominal tool diameter and feed rate can be observed. At higher tool diameters and feed rates, the relationship appears linear, whereas at lower feed rates, the dependency becomes highly nonlinear.

### Detailed Analysis of Drilling Process Parameters by Decision Tree

The conducted analyses revealed that the influence of selected technological and tool factors on torque variation is complex, further influenced by the machined material. A comprehensive approach is necessary to fully understand this relationship. A detailed analysis of the drilling process, focusing on torque variation, was performed using machine learning, specifically decision trees, with the CERT method [33] selected for tree construction. Based on experimental measurements, the constructed decision tree achieved an adjusted coefficient of determination of 99.460% (RMSE = 0.1618, MSE = 0.0262, MAD = 0.1201, MAPE = 0.0258) with a total of 118 nodes. The key factor affecting torque variation, with an average torque value of 5.557 ± 0.112 Nm across the dataset, was identified as the nominal tool diameter. Considering the mean values of torque (*M_c_*), two statistically distinct groups (*p* < 0.000) were identified. The first group with nominal tool diameters of D = 8.0 mm and D = 8.9 mm exhibited a mean torque of 3.822 ± 0.077 Nm; the second group with *D* ranging from D = 10.0 mm to D = 12.0 mm had a mean torque of 6.779 ± 0.134 Nm.

The following analysis focuses on these two defined groups. At lower nominal tool diameters (8.0 mm and 8.9 mm), the influence of feed rate behaves differently. For low feed rates (0.090 and 0.130 mm·rev^−1^), the mean torque value is 2.495 ± 0.101 Nm, whereas for feed rates above 0.175 mm·rev^−1^, the mean torque increases to 4.414 ± 0.065 Nm, with a statistically significant difference (*p* < 0.000). The effect of material on torque variation becomes apparent at *f_n_* = 0.130 mm·rev^−1^, where the mean torque for drilling 16MnCr5 steel is 2.715 ± 0.070 Nm, and for C45 steel, it reaches 3.233 ± 0.070 Nm, with a statistically significant difference (*p* < 0.000). At minimum feed rates (*f_n_* = 0.090 mm·rev^−1^), the effect of material varies depending on cutting speed. For cutting speeds between *v_c_* = 80.21 and 115.00 m·min^−1^, the mean torque for drilling 16MnCr5 is 1.940 ± 0.052 Nm, whereas for C45 steel, it is 2.111 ± 0.057 Nm. However, at higher cutting speeds (*v_c_* = 134.50 to 149.79 m·min^−1^), the trend reverses, with mean torque values of 2.155 ± 0.061 Nm for 16MnCr5 and 1.833 ± 0.049 Nm for C45. For feed rates above 0.130 mm·rev^−1^, the effect of material on torque variation is influenced by the helix angle (*ω_r_*), where an increase in *ω_r_* leads to a reduction in torque.

In the second group, which includes nominal tool diameters ranging from D = 10.00 to 12.00 mm, torque variation is primarily influenced by feed rate. The feed rates can be categorized into two ranges: *f_n_* = 0.090 to 0.175 mm·rev^−1^, where the mean torque is 5.945 ± 0.084 Nm, and *f_n_* = 0.220 to 0.260 mm·rev^−1^, where the mean torque increases to 9.269 ± 0.173 Nm. At a feed rate of *fₙ* = 0.090 mm·rev^−1^, the nominal tool diameter becomes a key factor in torque variation. For D = 10.0 mm and 11.10 mm, the mean torque is 2.871 ± 0.081 Nm, with C45 steel showing a mean torque of 2.711 ± 0.007 Nm and 16MnCr5 steel showing 3.031 ± 0.051 Nm. When the nominal tool diameter increases to D = 12.0 mm, the mean torque rises to 3.332 ± 0.043 Nm for C45 and 3.955 ± 0.073 Nm for 16MnCr5, indicating that torque increases with tool diameter, with a more pronounced effect when drilling 16MnCr5 steel. At feed rates of *f_n_* = 0.130 and 0.175 mm·rev^−1^, nominal tool diameter remains a decisive factor in torque variation, leading to the formation of two distinct groups. The first group consists of tools with D = 10.0 mm, where the effect of material varies depending on the specific feed rate value. At a feed rate of *f_n_* = 0.130 mm·rev^−1^, the mean torque value for C45 steel is 4.599 ± 0.046 Nm, while for 16MnCr5 steel, it is 4.110 ± 0.012 Nm. However, at *f_n_* = 0.175 mm·rev^−1^, the effect of material on torque variation depends on both the point angle (*ε_r_*) and the helix angle (*ω_r_*). When the point angle is *ε_r_* = 134.50° or 145.10°, an increase in the helix angle leads to a decrease in conditional torque values, with the material effect becoming less pronounced at higher *ω_r_* levels. Interestingly, at *ω_r_* = 32.80° and 35.00°, the torque value for C45 steel (4.619 ± 0.014 Nm) is lower than that for 16MnCr5 steel (4.729 ± 0.016 Nm), while at *ω_r_* = 25.00° and 30.00°, the trend reverses, with C45 showing a higher torque (5.185 ± 0.017 Nm) compared to 16MnCr5 (4.919 ± 0.009 Nm). This confirms the previous observation that increasing the helix angle reduces the torque values. At higher feed rates (*f_n_* = 0.220 and 0.260 mm·rev^−1^), the dominant factor remains the nominal tool diameter (*D*). For D = 10.00 mm and 11.10 mm, a significant material effect is observed only at *f_n_* = 0.260 mm·rev^−1^, where the mean torque for C45 (8.947 ± 0.111 Nm) is higher than for 16MnCr5 (7.930 ± 0.016 Nm). At a nominal tool diameter of *D* = 12.00 mm and a feed rate of *f_n_* = 0.220 mm·rev^−1^, there is no significant difference in torque between C45 (9.583 ± 0.177 Nm) and 16MnCr5 (9.149 ± 0.203 Nm). However, at *f_n_* = 0.260 mm·rev^−1^, the mean torque for C45 (12.104 ± 0.156 Nm) is statistically significantly higher than for 16MnCr5 (10.659 ± 0.155 Nm).

The conducted analyses indicate that the influence of selected technological and tool factors on torque variation during drilling is highly complex, requiring a detailed approach to fully understand its behaviour. Additionally, the results confirm that the machined material plays a significant role in torque variation. Although its direct contribution appears relatively small (1.430%), it has a fundamental impact on the way selected input variables interact and influence torque behaviour.

## 4. Conclusions

The main objective of this study was to conduct an experimental analysis of the influence of key technological (*f_n_*, *v_n_*) and tool-related (*D*, *ε_r_*, *α*_0_, *ω_r_*) factors on torque variation (*M_c_*) during the drilling process of two materials: C45 steel (R_m_ = 740.500 ± 1.445 MPa, HV10 = 221.500 ± 3.023) and 16MnCr5 steel (R_m_ = 740.500 ± 1.447 MPa, HV10 = 221.500 ± 3.023). Based on an extensive experimental investigation, the following conclusions can be drawn:

- the influence of material on torque variation (*M_c_*) accounts for 1.430%, yet its impact is statistically significant (*p* < 0.000)
- the dominant factor influencing torque variation (*M_c_*) is the nominal tool diameter (*D*), contributing 53.552%
- the influence of feed rate (*f_n_*) on torque variation (*M_c_*) is 36.263% and is statistically significant (*p* < 0.000)
- the point angle (*ε_r_*) contributes 1.189% to torque variation (*M_c_*) and is statistically significant (*p* < 0.000)
- the helix angle (*ω_r_*) contributes 0.310% to torque variation (*M_c_*) and is statistically significant (*p* < 0.000)
- the influence of cutting speed (*v_c_*) on torque variation (*M_c_*) was not confirmed (*p* = 0.354).

In addition to these general conclusions, the mechanical properties of the machined material also influence the effect of individual selected technological and tool factors on torque variation (*M_c_*). When drilling C45 steel, the nominal tool diameter (*D*) contributes 50.115% to torque variation (*p* < 0.000), while the influence of feed rate (*f_n_*) is 41.603% (*p* < 0.000); the point angle (*ε_r_*) contributes 0.486% (*p* < 0.000) and the helix angle (*ω_r_*) accounts for 0.680% (*p* < 0.000). Conversely, when drilling 16MnCr5 steel, the influence of nominal tool diameter (*D*) increases to 60.373% (*p* < 0.000). At the same time, compared to C45 steel, the influence of feed rate decreases to 31.480% (*p* < 0.000), while the point angle’s effect increases to 2.562% (*p* < 0.000). The helix angle (*ω_r_*) contributes only 0.098% to torque variation for 16MnCr5 steel but remains statistically significant (*p* = 0.002).

The analysis conducted using a machine learning approach validated these conclusions while also revealing the complex interdependencies between tool and technological factors in relation to torque during drilling. This underscores the need for a more comprehensive investigation of the drilling process, extending beyond the scope of this study to include both conventional and unconventional materials commonly used in industry. Finally, it is important to acknowledge the limitations of this study. Due to the inherent constraints of experimental research, the findings cannot be universally generalized, as their applicability is restricted to the specific experimental conditions and materials used.

## Figures and Tables

**Table 1:** Chemical composition of materials used in the experiments.

Element	Chemical Composition in Weight Percent [wt%]	
	C45	16MnCr5
C	0.5870	0.1960
Mn	0.7620	1.6000
Si	0.3530	0.2030
P	0.0117	0.0063
Si	0.0048	0.0238
Cr	0.2160	0.9450
Ni	0.0280	0.0944
W	0.0070	0.0000
Mo	0.0210	0.0220
V	0.0032	0.0017
Cu	0.0297	0.1410

**Table 2:** Levels of varied tool and technological factors.

Code	Factor	Unit	Factor Level				
			1	2	3	4	5
*x*_1_	*D*	[mm]	8.00	8.90	10.00	11.10	12.00
*x*_2_	*ε_r_*	[°]	130.00	133.30	137.50	141.70	145.00
*x*_3_	*ω_r_*	[°]	25.00	27.20	30.00	32.80	35.00
*x*_4_	*f_n_*	[mm·rev^−1^]	0.09	0.13	0.175	0.22	0.26
*x*_5_	*v_c_*	[m·min^−1^]	80.21	95.50	115.00	134.50	149.79

Note: *D*—nominal drill diameter, *ε_r_*—tool’s point angle, *ω_r_*—helix angle, *f_n_*—revolution feed, *v_c_*—cutting speed.

## Data Availability

The original contributions presented in this study are included in the article. Further inquiries can be directed to the corresponding author.
